# Use cases for genetic epidemiology in malaria elimination

**DOI:** 10.1186/s12936-019-2784-0

**Published:** 2019-05-07

**Authors:** Ronit Dalmat, Brienna Naughton, Tao Sheng Kwan-Gett, Jennifer Slyker, Erin M. Stuckey

**Affiliations:** 1 Department of Epidemiology, University of Washington, Seattle, WA USA; 2 Department of Global Health, University of Washington, Seattle, WA USA; 3 Department of Health Services, University of Washington, Seattle, WA USA; 4 Strategic Analysis Research and Training Center, University of Washington, Seattle, WA USA; 5 Bill and Melinda Gates Foundation, Seattle, WA USA

**Keywords:** Malaria, Use case, Genetic epidemiology, Drug resistance, NMCP, Gene flow, Eradication, Surveillance, Policy development, Transmission

## Abstract

**Background:**

While traditional epidemiological approaches have supported significant reductions in malaria incidence across many countries, higher resolution information about local and regional malaria epidemiology will be needed to efficiently target interventions for elimination. The application of genetic epidemiological methods for the analysis of parasite genetics has, thus far, primarily been confined to research settings. To illustrate how these technical methods can be used to advance programmatic and operational needs of National Malaria Control Programmes (NMCPs), and accelerate global progress to eradication, this manuscript presents seven *use cases* for which genetic epidemiology approaches to parasite genetic data are informative to the decision-making of NMCPs.

**Methods:**

The use cases were developed through a highly iterative process that included an extensive review of the literature and global guidance documents, including the 2017 World Health Organization’s Framework for Malaria Elimination, and collection of stakeholder input. Semi-structured interviews were conducted with programmatic and technical experts about the needs and opportunities for genetic epidemiology methods in malaria elimination.

**Results:**

Seven use cases were developed: Detect resistance, Assess drug resistance gene flow, Assess transmission intensity, Identify foci, Determine connectivity of parasite populations, Identify imported cases, and Characterize local transmission chains. The method currently used to provide the information sought, population unit for implementation, the pre-conditions for using these approaches, and post-conditions intended as a product of the use case were identified for each use case.

**Discussion:**

This framework of use cases will prioritize research and development of genetic epidemiology methods that best achieve the goals of NMCPs, and ultimately, inform the establishment of normative policy guidance for their uses. With significant engagement of stakeholders from malaria endemic countries and collaboration with local programme experts to ensure strategic implementation, genetic epidemiological approaches have tremendous potential to accelerate global malaria elimination efforts.

**Electronic supplementary material:**

The online version of this article (10.1186/s12936-019-2784-0) contains supplementary material, which is available to authorized users.

## Background

In recent years, public health campaigns have achieved major reductions in malaria morbidity and mortality globally, with incidence decreasing by 18% globally between 2010 and 2016 [1].

As many countries accelerate their efforts towards elimination (World Health Organization (WHO) E-2020 Initiative) and the world works towards global eradication (Sustainable Development Goal (SDG) 3.3), programmatic priorities are changing and new data needs emerging. Traditional epidemiological approaches to surveillance analysing the reported clinical-based incidence data available through the routing system and/or cross-sectional studies of prevalence in a population were previously sufficient for National Malaria Control Programmes (NMCPs), whose aim is to contain and estimate the burden of infection within its borders. However, higher resolution information—i.e., more detailed, comprehensive, accurate, and informative—about parasite transmission dynamics will be necessary to target interventions or even predict when, where, and which interventions will be most effective to eliminating remaining reservoirs of infection.

Genetic epidemiology is a discipline that deals with the role of genetic factors and their interaction with environmental factors in the occurrence of disease in populations [2]. Analysing parasite genetics has enormous potential to aid both NMCP efforts at elimination as well as international efforts toward eradication [3, 4]. To date the genetic epidemiology field has primarily focused on applied research. For example, the use of molecular markers of anti-malarial drug resistance has for some time been used to guide the efficacy studies that define treatment policy; more recently, the detection of parasites mutant for histidine-rich protein 2/3 (pfhrp2/pfhrp3) gene highlighted the need to develop rapid diagnostic tests (RDTs) with alternative targets of detection, and to provide countries with guidance on the implications for case management [5]. A global scientific network providing framework for generating, integrating and sharing malaria parasite and vector genetic and genomic data is available through the Malaria Genomic Epidemiology Network (MalariaGEN) [6].

**Fig. 1:**
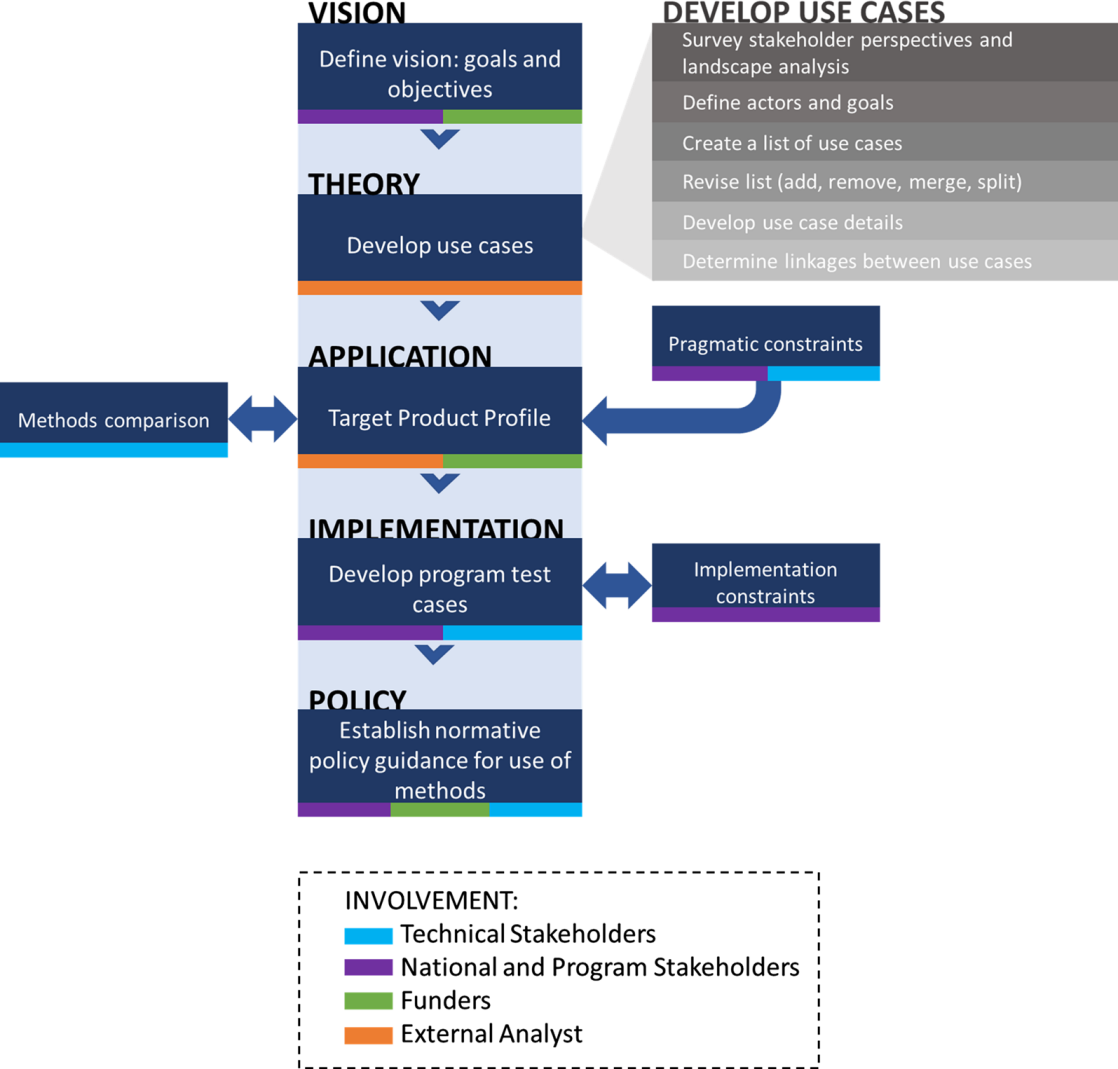
A roadmap for realizing the potential of genetic epidemiology for malaria elimination. The development of use cases is just one step in the broader pathway necessary for the effective global use of novel approaches to achieve the vision of a malaria-free world. This pathway illustrates the steps through which technical and programme stakeholders and funders can be collectively engaged to ensure the application and implementation of genetic epidemiology approaches to address programme needs and elimination priorities. Use cases represent the link between information sought by programme decision-makers and information provided by the interpretation of parasite genetics. Each use case may demand similar or different technological capabilities, which are detailed in a Target Product Profile (TPP) developed for each use case. A TPP serves as a benchmark by which to compare technological platforms and analysis methods against each other. The TPP and methods comparison steps indicate which platforms should be used for pilot testing in real-world programmatic settings. Program test cases ultimately inform the establishment of normative policy guidance for global implementation

Given this vision for genetic epidemiology approaches in NMCP elimination strategies and the need for WHO normative policy guidance to effect global implementation, there is an emerging need to clearly articulate the uses of these methods and how they can help advance programmatic and operational needs of NMCPs as well as accelerate progress toward SDG 3.3. To bridge the divide between research and implementation, *use cases* are a means to align research, technology development, and public health efforts using genetic epidemiology methods in support of NMCP priorities. Use case analysis is an analytic framework commonly employed in software and business industries to assess the ways a system can be used and illustrate its applications in real world contexts [7]. A use case provides a functional description of a system, as well as a framework to develop derivations of test cases based on available technologies. They are then used to inform the application and implementation of new technologies through ongoing engagement with stakeholders. Other health domains have deployed a use case methodology for emerging technologies, including soil-transmitted helminth diagnostics [8] and unmanned aerial vehicles [9]. This report represents a novel application of this methodology to genetic epidemiology and is intended to launch a cohesive collaboration by genetics researchers and public health programmes towards the use of these approaches (Fig. 1).

## Methods

To begin, a review was conducted of literature regarding genetic epidemiology and malaria elimination strategies. The literature review engaged an exploratory search strategy in both published and grey literature databases such as the MESA research hub [10], starting with key search terms, and allowing those findings to inform the next cycle of search terms and contacts in a cascade search. Initial search terms included malaria transmission, genetic epidemiology, genetic sequencing, and population genetics.

To inform and complement the literature review, between March and September 2017, individual in-depth interviews were conducted with 15 international stakeholders identified through the review—including malaria disease experts, laboratory and field researchers, programme implementers, mathematical modellers, policymakers, and donors for their input as to the needs and opportunities for genetic information in malaria elimination. Interviews were conducted in person or over the phone using a semi-structured interview guide (Additional file 1).

The development of the list of use cases was informed by the critical review of the literature and informational interviews with experts. The use cases were aligned to activities specifically described in Pillars 2 and 3 of the updated 2017 WHO Framework for Malaria Elimination [11], stemming from the WHO Global Technical Strategy [12]. These two pillars comprise activities for which genetic epidemiology applications have the most promising applications: surveillance, case investigation, and stratification of areas for targeted interventions to reduce transmission and accelerate elimination. Activities falling outside the WHO framework, for example using genetic techniques to guide product development such as rapid diagnostic tests (RDTs), were considered out of scope. This use case framework is modeled on an early draft of use cases developed by a working group of soil-transmitted helminths diagnostic experts published last year [8]. Finally, the categorization and content of the use cases was iteratively developed and revised by sharing drafts with previously interviewed experts.

## Results

Seven use cases are presented where genetic epidemiology approaches are informative to decision-making within the efforts of NMCPs. The use cases are intended to be technology-agnostic such that they may be generalizable across many endemic settings with different epidemiological characteristics (e.g. disease prevalence, drug resistance incidence, and transmission intensity), as well as applicable to emerging genetic epidemiology methods.

### Components of a malaria use case

Use cases are designed to align multiple scientific fields related to malaria control and eradication under a singular goal, incorporating research and programme perspectives. The structure of a use case delineates the conditions under which the method or tool would be applicable and useful. The content of each use case, presented here, is organized into three components: description, pre-conditions, and post-conditions.

The description section conveys, briefly, the objective sought by use of the method—what information the genetic epidemiology method is providing—as well as the current method used to achieve that objective in the absence of a genetic method. Pre-conditions describe the type of case detection (active, passive, or reactive), prevalence of infection (high, moderate, low, and very low), and focus type (active, residual non-active, and cleared). These categorizations of epidemiological characteristics are all aligned to those used in the WHO Framework for Elimination and use the same definition. Sampling frame (representative or dense) is also listed as a pre-condition to depict the degree to which the target population needs to be sampled for a genetic method to be informative. Finally, the population level of implementation (e.g. individual or focus) is described. These vary across use cases and serve to provide an orientation to the objectives. Post-conditions focus on the consumers of information and depict how the data will be identified, utilized, and presented. This includes the potential actions informed by the results of the genetic method, presentation of that output, and the ideal timeframe between sample collection and delivery of data analysis on which the information would be available from the method in order to be informative to programmatic decision-making.

For some of the use cases described here, genetic methods have been applied in a malaria control or elimination setting and described in the literature (Use cases 1, 2, and 6); methods for other use cases have not yet been applied programmatically (Use cases 3, 4, 5, and 7). Use cases are provided in Table 1 and described in short narratives below, with one or more example methods, if available.

**Table 1:** Use cases for genetic epidemiology in malaria elimination

	Description			Pre-conditions					Post-conditions		
	Description	Current method or indicator	Population unit for implementation of analysis	Case detection	Prevalence^a^	Focus type	Sampling frame	Informatics; priors needed	Potential action informed	Presentation of output	Time to information (ideal)
1. Detect resistance	Assess the prevalence/frequency of molecular markers associated with antimalarial drug resistance	PCR-based testing (currently research focused)	Individuals	Passive or active	High to very low	Active	Representative	Database of resistance alleles in local population; ability to identify possible new parkers	Intervention selection, treatment guidelines, surveillance	Quantitative, geospatial maps	Rapid (< 1 week)^b^
2. Assess drug resistance gene flow	Monitor and predict the spread of genes conferring drug resistance within and among regions and parasite populations	Treatment efficacy surveys at variable frequencies	Multiple foci (e.g. Areas where administration of drugs is a major component of control effort)	Active	High to very low	Active	Representative	Reference distribution of resistance alleles; model for gene flow with genetic data input	Intervention selection, treatment guidelines, surveillance	Quantitative, geospatial maps, phylogenies	Monthly^b^
3. Assess transmission intensity	Stratify regions according to transmission intensity in the area population; monitor interventions and epidemics	Surveillance	Focus	Passive or active	Low to very low	Active	Representative^c^	Reference distribution of parasite diversity; intensity model using a genetic data input	Intervention selection and evaluation, deployment of resources	Quantitative, qualitative, geospatial maps	Monthly
4. Identify foci	Identify focal areas of high diversity and clusters of infections	Surveillance, case investigation	Geographic area of interest (e.g. Area with unknown distribution of foci or hotspots)	Passive, active, reactive	Moderate to very low	Active to residual non-active	Representative	Algorithm to integrate case detection with geographic and population characteristics	Intervention selection, surveillance, deployment of resources	Phylogenies, geospatial maps	Fast (< 1 month)
5. Determine connectivity of parasite populations	Assess degree to which transmission is linked among regions due to parasite population linkages	Migration data (often produced via modeling)	Multiple foci across a region, country, or continent (e.g. Areas where parasite populations may be linked due to human or parasite migration)	Active	High to very low	Active to residual non-active	Representative	Reference distribution of parasite diversity and human migration patterns; model for parasite flow with genetic data input	Intervention selection and evaluation, deployment of resources	Geospatial network maps	Annual
6. Identify imported cases	Discriminate between indigenous vs. imported cases (sources and sinks)	Travel surveys	Individuals	Passive or reactive	Very low to zero	Active to residual non-active	Dense	Reference distribution of local parasite diversity; high coverage case surveillance	Intervention selection and evaluation, deployment of resources, surveillance, case investigation; certify elimination	Quantitative, phylogenies, network maps	Rapid (< 3 days)^d^
7. Characterize local transmission chains	Distinguish contributions factors (e.g. seasonality, migrants, asymptomatics, and highly infectious individuals) to ongoing transmission patterns; certify elimination	Case investigations	Focus with limited transmission	Passive or reactive	Low to very low	Active to residual non-active	Dense	Reference distribution of parasite diversity; models engaging geospatial and/or network analysis to distinguish chain length	Intervention selection and evaluation, deployment of resources, surveillance, case investigation; certify elimination	Quantitative, phylogenies, network maps	Fast (< 1 month)

^a^While this information may be applicable at higher levels of prevalence, current methods suggest that genetic information is most useful in areas of low transmission that are progressing to zero

^b^Treatment regimen may not be adaptable in this timeframe, depending on drug availability

^c^Higher coverage is required for populations with more complex substructure or high parasite relatedness

^d^This period is an estimate of how quickly a decision needs to be made about whether a full case investigation should be conducted

These use cases are designed to align the efforts of multiple scientific fields towards the shared goal of malaria elimination, incorporating both research and programme perspectives. The content of each use case presents the conditions under which the method or tool is applicable and how it is useful.

### Use case #1: Detect resistance

This use case describes a method that identifies drug resistant parasites within an individual with high sensitivity and specificity, regardless of the presence of symptoms or treatment failure, in a high to very low prevalence setting. Treatment efficacy surveys are currently used to assess population drug resistance but are time-consuming, costly, and may occur with insufficient frequency to inform decision-making. At a population level, these methods, compared to traditional methods, would serve to estimate resistance prevalence more accurately in a country and accelerate the identification of population trends in the emergence of resistance. Numerous genotyping methods have been developed for detecting particular genetic markers in individual patient samples; nested and real-time polymerase chain reaction (PCR) are used widely in a variety of country settings. Other newer methods still in development include: point-of-care resistance testing with loop-mediated isothermal amplification [13, 14] and next generation sequencing (NGS) of single nucleotide polymorphism (SNPs) [15]. Each approach has advantages and limitations described in the literature [16–20].

### Use case #2: Assess drug resistance gene flow

The method depicted in this use case provides information about how resistance genes are spreading in a population of parasites in a setting with high to very low prevalence. The information provided by the use case enables identification of linked populations and predictive modelling of drug-based interventions. Methods for population genetic analysis have been employed to understand the interaction between gene flow and patterns of resistant marker prevalence in several geographies including Ethiopia [21] and Cambodia [22], as well as the dispersal of resistance haplotypes in Eastern Africa and the Democratic Republic of Congo [23]. NGS technologies are also emerging as a tool for drug resistance surveillance [24]. This use case is linked to the method described by Use case 1, as it would require aggregation of information collected by Use case 1, along with additional analysis to predict emergence or dissemination of drug resistance into new areas. Application of this use case is most applicable at a regional rather than national level in settings where national malaria programmes interact, such as the Asia Pacific Malaria Elimination Network.

### Use case #3: Assess transmission intensity

This use case describes a method that allows decision-makers to stratify regions according to transmission intensity in low and very low prevalence settings and monitor the effect of interventions. While current methods use surveys and weekly case counts to measure changes in prevalence and incidence, they are unable to measure genetic signatures indicative of changes in transmission intensity at low prevalence levels, such as changes in parasite population structure. For example, stagnation or increasing case counts might suggest an intervention is failing, even though the resurgence could be due to rising transmission rates from a source not exposed to the intervention. Additionally, for countries approaching elimination, this use case describes a method that can assess changes in transmission at low prevalence (< 2%), where prevalence estimates and case counts are imprecise due to geographical heterogeneity and are highly dependent on the population’s access to health care. SNP barcodes have been used to define transmission patterns in populations in many countries, including Senegal [25], Malawi [26], Zambia [27], Cambodia [28], Panama [29], and through longitudinal tracking in Papua New Guinea [30]. Additional investigation is needed to advance and validate methods for the translation of parasite genetic diversity into a measurement of parasite transmission.

### Use case #4: Identify foci

This use case defines a method that enables the identification and stratification of foci according to receptivity and transmission intensity, as recommended by WHO guidelines, in moderate to very low prevalence settings [12]. Specifically, the method measures how closely related parasites are to each other and estimates transmissibility of parasites within different groups. Currently, foci may be inferred using evidence from geospatial surveillance and case investigations, but only a genetic method can definitively link cases based on parasite genetic relatedness and therefore validate current methods for foci identification [31]. A method for identifying foci, especially in very low transmission settings, is likely to be linked to the method described by Use case 3 because transmission intensity is a characteristic used in foci stratification. In two examples, SNP-genotyping analysis generated by PCR-based assays was used to characterize the Makira region of Madagascar as a hotspot [32] and assays of microsatellites were used to attribute the resurgence of *Plasmodium vivax* malaria in Greece to specific villages with foci reactivated by imported malaria via migrant agricultural workers [33]. However, it is unclear what conditions and geographic scale are necessary to enable the identification of transmission foci that are stable over time using genetic surveillance.

### Use case #5: Determine connectivity of parasite populations

The method described in this use case determines how parasite populations are linked across various geographic regions with high to very low prevalence and enables a quantitative assessment of how much of transmission in one region is due to contributions from transmission in another. The method is related to the method presented in Use case 2, though without the specificity of anti-malarial resistance. Therefore, the two use cases may engage similar methods of measurement and analysis and are both relevant at a regional level. Estimates of how parasite populations are linked geospatially may be approximated from human migration data [34], but this use case proposes a method with higher spatiotemporal resolution. Gene flow has been characterized by a range of analytic techniques using SNP genotype data from nested PCR in Papua New Guinea [35] and at the Thai-Myanmar border [36], and genome-wide sequencing in Mauritania, among other countries [37]. However, these methods vary in their resolution to assert connectivity and additional evidence is needed to validate the associations between genetic patterns of population structure and human migration. An estimate of parasite population connectivity is an essential parameter for epidemiological models of malaria, which may be useful for optimizing elimination strategies [38].

### Use case #6: Identify imported cases

This use case describes a method for discriminating between indigenous and imported cases in areas with low or zero prevalence by measuring how similar a parasite is to a population of parasites currently or previously endemic to a geographically relevant area. In countries at or approaching zero cases, the method described in this use case provides a more definitive determination than can be offered through the use of travel surveys and case investigations. Several genetic methods have been demonstrated for this use cases in the literature, especially in countries at or approaching elimination that need to confirm whether a reported case or cluster of cases is due to local transmission or importation in order to maintain their elimination progress. Two high profile examples of such a method in use include: a cluster of cases in Puerto Rico confirmed to be imported from the Dominican Republic [39] and an outbreak of *Plasmodium falciparum* malaria among United Nations peacekeeping soldiers in Guatemala that was attributed to exposure when they were stationed in the Democratic Republic of Congo [40]. Optimization of these methods will be required to generate information useful in real-time scenarios.

### Use case #7: Characterize local transmission chains

The method described by this use case will be linked to the one proposed in Use case 6, but with higher resolution to distinguish the contribution of specific sources to ongoing or recurring transmission in an area with low to very low prevalence. The method will provide a high-resolution snapshot of how cases are related to each other—a result definitively attainable only through a genetic method. Additionally, the method will need to incorporate both epidemiological and genetic information, as well as complex modeling methods to provide a snapshot of what is contributing to ongoing transmission. This use case is the most abstract in its conceptualization because methods for this purpose are mostly theoretical or have been modeled in specific research contexts [41]. Furthermore, a method for this use case will require a well characterized parasite population (historical and dense surveillance sampling) for the method to be applicable in that region. Few, if any, malaria endemic countries in the world have sufficiently historical and consistent genetic surveillance of their local parasite populations to contextualize a method for this use case.

## Conclusion

The use cases presented in this article represent a novel application of a common business technique, applied elsewhere in fields of public health and software, and here to address the research-implementation gap in malaria. They have already been used to guide discussions at two international meetings focusing on the interactions between NMCP representatives and genome scientists, hosted by the Harvard T. Chan School of Public Health and the Massachusetts Institute of Technology Broad Institute (July 2017), and the Mahidol-Oxford Research Unit (January 2018).

Successful development and implementation of genetic epidemiological methods, through the steps depicted in Fig. 1, will require continued buy-in and information sharing between technical and programmatic experts, as well as resource investments that advance the capacity of NMCPs to involve these methods in their activities. This will be especially important in the translation of these use cases into the technical specifications needed for Target Product Profile (TPPs) that are directly responsive to specific NMCP information needs, including specific endemic settings and parasite-species specific requirements. NMCPs should drive the design of TPPs, and it is expected that different use cases will be prioritized by different countries and regions, depending on both epidemiological and operational contexts. While many of the use cases span a range of transmission intensities, there is a need to validate use cases, and the methods themselves, in higher burden settings. For example, routine monitoring for emergence of known antimalarial resistance markers in *P. falciparum* may be demanded by NMCPs in sub-Saharan Africa, while high resolution information about the spread of antimalarial resistance in *P. vivax* populations would be more useful in the Greater Mekong Subregion, Latin America, and other regions with endemic *P. vivax*. The development of TPPs may suggest opportunities for developing genetic epidemiology methods that address multiple use cases.

Additional implementation constraints and limitations of these new methods will also need to be explored in pragmatic test cases led by NMCPs to ensure these methods are cost effective in achieving elimination targets. Feasibility of each use case per country context will depend on factors including, but not limited to, the analytical capacity available, what sampling frame is logistically and financially feasible for ascertaining useful information at different levels of geographic granularity, the strength of the routine surveillance system, and availability and throughput capacity of laboratory technologies. There are pros and cons for NMCPs to use genetic approaches to answering epidemiological questions, and it is necessary to conduct a cost–benefit analysis for each use case. Significant involvement of programme experts and malaria-endemic country stakeholders will be required to help genetic technology developers understand where methods could be standardized for cost and supplement or perhaps even existing programme activities. This will help NMCPs understand whether existing epidemiological methods are sufficient in their country compared to opting for combining those methods with a genetic epidemiology approach, whether it is preferential to conduct this analysis in-country or in regional or global centers, and how best to integrate genetic epidemiology data into existing analysis for their decision-making [42].

It is worth noting that as this framework focuses on use cases applicable to questions NMCPs would ask, there are many more applications of genetic epidemiology for a research agenda than the use cases listed here. As one example, genotyping data can help us understand how long parasites persist, a critical element for assessing the infectious reservoir and from there for predicting the impact of interventions. In addition, having a representative sample of parasites globally would allow for prospective screening of drug resistance candidates to identify emerging resistance before countries start to observe treatment failure. A research agenda is beyond the scope of this article and would be a very welcome way of focusing efforts of the research community. Finally, these use cases can be extended to include vector genomics as the genomes of more vector species are sequenced and new methods emerge to detect and predict insecticide resistance emergence and spread.

By providing a framework for the application of genetic epidemiology approaches, these use cases can launch the process towards developing normative guidance for their appropriate use in global malaria eradication, a key element for any new tool or method to get to scale.

## Additional file

**Additional file 1.** Technical and programmatic interview guides developed by the START center used in the semi-structured interviews with experts in the field.

## Abbreviations

HRP2/HRP3: histidine-rich protein 2/3
NMCP: National Malaria Control Programme
NGS: next-generation sequencing
PCR: polymerase chain reaction
RDT: rapid diagnostic test
SDG: Sustainable Development Goals
SNP: single nucleotide polymorphism
TPP: Target Product Profile
WHO: World Health Organization
