# Supplementary material for: Use cases for genetic epidemiology in malaria elimination
Source: Malar J. 2019 May 7;18:163. doi: 10.1186/s12936-019-2784-0 (PMC6503548; doi:10.1186/s12936-019-2784-0)
Supplement: Supplementary file 1 — Additional file 1. Technical and programmatic interview guides developed by the START center used in the semi-structured interviews with experts in the field. [file 12936_2019_2784_MOESM1_ESM.docx]

**START Center** | **Genomic Technology Expert Interviews**

***Interview Guide***

| **Framing** | |
| --- | --- |
| **Interviewee** | *[Name, Title]* |
| **Interviewing Team** | *[Names, Titles]* |
| **Interview Style** | Qualitative interviews, free-form, open-ended questions and prompts to elicit detailed responses. |
| **Introduction Narrative (by Interviewing Lead)** | *We are a team from the University of Washington’s Strategic Analysis and Research Training Center (known as the START Center). The START Center provides research and strategic support to the Bill & Melinda Gates Foundation (BMGF).*  *In coordination with the Malaria team at BMGF, we are investigating how genomic technologies can inform national malaria control programs and global malaria eradication efforts. For one of our objectives, we have been asked to build use cases of next generation genomic tools in malaria control. We hope to focus our discussion today around your knowledge of genomics in malaria control efforts and experience in developing the technology to track malaria transmission and outbreaks. We will then use this information, our discussions with other experts, and extensive desk research to guide our approach to developing and validating use cases.* |
| **Questions** | |
|  | Please tell us a bit about your background and role in malaria genomics research and technology development. |
|  | There is limited publicly available information regarding the current technology on which you are working. Could you share more information about that with us, including the main value add of this technology?   1. *Prompt for further details: Information on development stage, uses, implementation setting* 2. *For those doing sequencing:*     1. *What does sequencing provide above and beyond genotyping?*    2. *Can you provide detailed information on the benefits and challenges of this approach?*    3. *Prompt for further details: Resistance mapping; discovery of new resistance genotypes* |
|  | In what kinds of settings do you see this technology being particularly useful? How is it useful epidemiologically, and how is it useful for country policy-makers and implementers?   1. *Prompt for further details: Looking for clarification of epidemic type (holoendemic, seasonal, sporadic), resistance trends, etc* |
|  | What in-country capacities are needed for this technology, including physical and human resources? Why?   1. *Prompt for further details: Including lab, physical human resources, surveillance, etc* |
|  | What are the main limitations to this technology, in terms of scientifically, and from the perspective of implementers? Why? |
|  | Are there any specific aspects of a setting or type of epidemic where you would not recommend this technology? Why? |
|  | How is your team working to address or mitigate these limitations/gaps/challenges?   1. *Prompt for further details: Looking to identify threshold of usage for this technology, which will help to inform our use case* |
|  | Do you have any suggestions for anyone else that would be useful to speak to, or specific documents to suggest? |
|  | Could you please share any additional suggestions or ideas to help guide the development of use cases for this technology? |
|  | Is there anything else you would like to add? |

**START Center** | **Program Expert Interviews**

***Interview Guide***

| **Framing** | |
| --- | --- |
| **Interviewee** | *[Name, Title]* |
| **Interviewing Team** | *[Names, Titles]* |
| **Interview Style** | Qualitative interviews, free-form, open-ended questions and prompts to elicit detailed responses. |
| **Introduction Narrative (by Interviewing Lead)** | *We are a team from the University of Washington’s Strategic Analysis and Research Training Center (known as the START Center). The START Center provides research and strategic support to the Bill & Melinda Gates Foundation (BMGF).*  *In coordination with the Malaria team at BMGF, we are currently researching how genomic technologies fit into national malaria control programs and global malaria eradication efforts. For one of our objectives, we have been asked to build use cases of next generation sequencing tools in malaria control. We hope to focus our discussion today around your knowledge of genomics in malaria control efforts and experience in developing the technology to track malaria transmission and outbreaks. We will then use this information, and our discussions with other experts, to develop these use cases.* |
| **Questions** | |
|  | Please tell us a bit about your background and role in malaria elimination and eradication, malaria genomics research, and usage of these this technology. |
|  | Amidst the varying settings (such as capacity and resistance trends) and priorities for epidemics (elimination, eradication, control, resistance control, surveillance), what are the main programmatic questions you have?   1. What are the main malaria programmatic questions you have as an implementer and/or policy-maker? 2. What tools do you currently have available? 3. What tools do you need to answer the main questions? 4. How do you in your work discern the correct tools? |
|  | With genotyping and sequencing, what are the respective value adds under ideal conditions, and in reality? |
|  | What are the main limitations to these technologies? Are there any specific aspects of a setting or type of epidemic where you would not recommend these technologies be employed?   1. *Prompt for further details: Including lab, physical human resources, surveillance, etc* |
|  | Are you able to identify ways to mitigate these challenges, or is this simply technology-dependent? |
|  | Are there any specific aspects of a setting or type of epidemic where you would not recommend these technologies? Why? |
|  | How is your team working to address or mitigate these limitations/gaps/challenges?   1. *Prompt for further details: Looking to identify threshold of usage for this technology, which will help to inform our use case* |
|  | As we mentioned above, we are working to develop use cases for these technologies, are there any recommendations you have for this, or specific uses of any of these technologies that you suggest we investigate further?   1. *Prompt for further details: Looking to identify options for use cases, and threshold of usage for this technology, etc* |
|  | Do you have any suggestions for anyone else that would be useful to speak to, or specific documents to suggest? |
|  | Could you please share any additional suggestions or ideas to help guide the development of use cases for this technology? |
|  | Is there anything else you would like to add? |
